# Association of single-nucleotide polymorphisms in dual specificity phosphatase 8 and insulin-like growth factor 2 genes with inosine-5′-monophosphate, inosine, and hypoxanthine contents in chickens

**DOI:** 10.5713/ab.23.0080

**Published:** 2023-06-23

**Authors:** Jean Pierre Munyaneza, Minjun Kim, Eunjin Cho, Aera Jang, Hyo Jun Choo, Jun Heon Lee

**Affiliations:** 1 Division of Animal and Dairy Science, Chungnam National University, Daejeon 34134, Korea; 2 Department of Bio-AI Convergence, Chungnam National University, Daejeon 34134, Korea; 3 Department of Applied Animal Science, College of Animal Life Science, Kangwon National University, Chuncheon 24341, Korea; 4 Poultry Research Institute, National Institute of Animal Science, Pyeongchang 25342, Korea

**Keywords:** Dual-specificity Phosphatase 8 (*DUSP8*), Insulin-like Growth Factor 2 (*IGF2*), Kompetitive Allele-specific Polymerase Chain Reaction (KASP), Korean Native Chicken, Polymerase Chain Reaction-Restriction Fragment Length Polymorphism (PCR-RFLP)

## Abstract

**Objective:**

This study aimed to identify the single-nucleotide polymorphisms (SNPs) in the dual-specificity phosphatase 8 (*DUSP8*) and insulin-like growth factor 2 (*IGF2*) genes and to explore their effects on inosine-5′-monophosphate (IMP), inosine, and hypoxanthine contents in Korean native chicken -red-brown line (KNC-R Line).

**Methods:**

A total sample of 284 (males, n = 127; females n = 157) and 230 (males, n = 106; females, n = 124) aged of 10 weeks old KNC-R line was used for genotyping of *DUSP8* and *IGF2* genes, respectively. One SNP (rs313443014 C>T) in *DUSP8* gene and two SNPs (rs315806609A/G and rs313810945T/C) in IGF2 gene were used for genotyping by polymerase chain reaction-restriction fragment length polymorphism (PCR-RFLP) and KASP methods, respectively. The Two-way analysis of variance of the R program was used to associate DUSP8 and IGF2 genotypes with nucleotide contents in KNC-R chickens.

**Results:**

The DUSP8 (rs313443014 C>T) was polymorphic in KNC-R line and showed three genotypes: CC, CT, and TT. The *IGF2* gene (rs315806609A/G and rs313810945T/C) was also polymorphic and had three genotypes per SNP, including GG, AG, and AA for the SNP rs315806609A/G and genotypes: CC, CT, and TT for the SNP rs313810945T/C. Association resulted into a strong significant association (p<0.01) with IMP, inosine, and hypoxanthine. Moreover, the significant effect of sex (p<0.05) on nucleotide content was also observed.

**Conclusion:**

The SNPs in the *DUSP8* and *IGF2* genes might be used as genetic markers in the selection and production of chickens with highly flavored meat.

## INTRODUCTION

Meat has long been consumed as food in various cultures [1–3]. Chicken meat has gained popularity globally due to its affordable price, low in fat, rich in protein, vitamins, and mineral contents [4–6]. Among various types of meat, poultry meat including chickens, is generally considered to be nutritious and healthier, and is preferred by meat consumers of all ages [5]. Indigenous livestock species, including chickens, are important genetic resources for the development of new breeds [7]. Korean native chickens (KNCs) are well adapted to the Korean climate [8] but they grow slowly, resulting in low productivity compared to broilers [9,10]. KNCs are subdivided into five lines based on the plumage color: white (KNC-W), black (KNC-B), yellow-brown (KNC-Y), gray-brown (KNC-G), and red-brown (KNC-R) [11–14].

To satisfy current meat preferences among consumers, which are based on quality, safety, and flavor, the National Institute of Animal Science (NIAS) of South Korea has attempted to develop a new high-quality KNC breed. The KNC-R line was selected for its higher meat quality, body weight, and contents of flavor-related compounds such as inosine 5′-monophosphate (IMP) compared to other KNC lines [15]. IMP is the most abundant nucleotide in meat [16] and is a major contributor to the taste and flavor in meat [17,18]. Inosine and hypoxanthine are IMP degradation products that also influence meat palatability [17]. The combination of IMP with glutamic acid or aspartic acid was reported to enhance the umami taste [17]. The nucleotide content of chicken meat is affected by the chicken breed, age, and sex, as well as the cooking method [11,14,16].

Flavor is among the most important meat quality parameters influencing consumer acceptability [6,19–23]. Numerous studies have shown that meat consumers prefer native chicken meat over broiler meat due to its higher content of taste-active compounds [24]. For example, meat consumers in South Korea, Japan, and Thailand were found to prefer meat from native chicken lines due to their flavor and texture [15,22], higher contents of nucleotides such as IMP and amino acids, including glutamic acid [22,24,25], and higher contents of flavor-related compounds [24], respectively. Thus, improving the content of taste-related compounds such as IMP in chicken meat through selection and breeding programs can improve the meat flavor and consumer acceptance [26].

The identification of candidate genes influencing meat quality traits such as flavor is a crucial step in the genetic improvement of the polygenic traits [27,28]. The involvement of genetic markers in the selection and breeding programs is an effective strategy because nucleotides have medium to high heritability [27–31]. For example, IMP was reported to have high heritability (0.51 to 0.69) in Taihe silkie chickens [26]. A genome-wide association study (GWAS) of meat quality traits in KNCs identified the dual-specificity phosphatase 8 (*DUSP8*) and insulin-like growth factor 2 (*IGF2*) as candidate genes influencing the nucleotide content in chickens [Unpublished manuscript]. To the best of our knowledge, this was the first study to explore the effects of *DUSP8* and *IGF2* genes on the content of IMP, inosine, as well as hypoxanthine in Korean native chickens.

Dual-specificity phosphatases (DUSPs) dephosphorylate the residues of both phosphoserine/threonine and phosphotyrosine on mitogen-activated protein kinases (MAPKs) [32–34]. MAPK signaling pathways are involved in cell proliferation, metabolism, motility, cell survival, cell death, apoptosis, cellular stress responses, cell differentiation, and immune responses [32,35,36]. DUSPs are important modulators and controllers of MAPK deactivation through dephosphorylation [33], and they play key roles in immune activation, brain function, and cell growth signaling [37]. DUSP8 expression has been reported to be higher in the adult human brain than in heart and skeletal muscle [34,38]. The DUSP8 polymorphism rs2334499 was found to be related to hypothalamic insulin resistance in men [38], whereas DUSP8 has been described as a gatekeeper for systemic glucose tolerance and hypothalamic insulin sensitivity [39].

IGF2 is a polypeptide hormone similar to insulin that performs many functions related to growth and the utilization of amino acids and glucose [39–42]. In humans, IGF2 impairment can lead to various metabolic disorders, including diabetes and obesity [43]. Several single-nucleotide polymorphisms (SNPs) of IGF2 have been associated with growth and meat quality traits in livestock species, including the growth rate and lipid metabolism of chickens [41]. The SNP of IGF2 was found to be significantly associated with body and carcass weight in 17-week-old Beijing You chickens [41].

In chickens, DUSP8 and IGF2 have been mapped to chromosome 5. The *DUSP8* gene has six exons and five introns (ENSGALG00000006647), whereas the *IGF2* gene has three exons and two introns (ENSGALG00000035282). Both DUSP8 and IGF2 are involved in glucose uptake; glucose provides energy in the form of ATP, which undergoes further degradation to produce IMP, followed by inosine and hypoxanthine [44,45]. We hypothesized that these genes could influence the nucleotide contents in chicken meat, thereby enhancing meat flavor; however, the effects of SNPs in DUSP8 and IGF2 on the IMP, inosine, and hypoxanthine contents of KNCs remain unclear. Therefore, the objective of this study was to identify SNPs in *DUSP8* and *IGF2* genes and to explore their effects on the IMP, inosine, and hypoxanthine contents in KNC-R chickens.

## MATERIALS AND METHODS

### Ethical statement

The protocol of this study was approved by the Animal Ethics Committee of Chungnam National University (202209A-CNU-141).

### Chicken samples and phenotype measurements

KNC-R chickens were obtained from the NIAS at the poultry research institute in Pyeongchang, South Korea, and maintained under consistent management and feeding conditions. Water was provided *ad libitum*. Genomic DNA was extracted from 284 chickens (127 males, 157 females) to examine SNPs in the *DUSP8* gene and from 230 chickens (106 males, 124 females) to examine SNPs in the *IGF2* gene. All chickens were slaughtered at 10 weeks of age, and were not fed for 12 hours but had access to water before slaughter, and were killed by conventional neck cut followed by bleeding, feather removing, and eviscerating in accordance with ethical guidelines. Breast meats were obtained from each carcass. Finally, a 100-g sample was collected from the breast of each chicken for nucleotide analysis.

### DNA extraction

Blood samples were collected from KNC-R chickens by the NIAS, transported to animal molecular genetics laboratory at Chungnam National University, and stored at −80°C until DNA extraction. A PrimePrep Genomic DNA Extraction Kit (GenetBio, Daejeon, Korea) was used to extract genomic DNA. The quality of genomic DNA was checked using spectrophotometry (NanoDrop 2000; Thermo Fisher Scientific, Waltham, MA, USA). DNA stocks were diluted with deionized distilled water to produce a working concentration of 25 ng/μL and stored at −20°C.

### Primer design and polymerase chain reaction amplification of *DUSP8* and *IGF2* genes

To identify SNPs, a pair of primers was designed to amplify the fragment (406 bp) of the chicken *DUSP8* gene containing the synonymous SNP of interest (rs313443014, C>T), and three pairs of primers were used to amplify all three exons of the *IGF2* gene. The primers used in this study were designed using the primer-BLAST tool (https://www.ncbi.nlm.nih.gov/tools/primer-blast) and synthesized by Bioneer Corp. (Daejeon, Korea). All primers used in this study are listed in Table 1.

Polymerase chain reaction (PCR) was performed in a 20-μL volume containing 2 μL of genomic DNA (25 ng/μL of chicken DNA), 1 μL each of the forward and reverse primers, 10 μL of HS Prime Taq Premix (2×) (GenetBio, Korea), and 6 μL of deionized distilled water. The conditions were: initial denaturation at 95°C for 3 min followed by 35 cycles of denaturation at 95°C for 30 s, annealing at 63°C for 45 s (for *DUSP8* gene), and extension at 72°C for 60 s; with a final extension at 72°C for 10 min (Table 1). Amplification was performed using a T100 Thermal Cycler (Bio-Rad, Hercules, CA, USA). A 2% agarose gel was used to visualize the products, and electrophoresis was run at 120 V for 30 min. The gels were visualized using an ultraviolet (UV) transilluminator.

### DNA purification, quality control, and sequencing for *IGF2* gene

We performed Sanger sequencing to identify SNPs in *IGF2* gene. The PCR products were purified using a PrimePrep PCR Purification Kit (GenetBio, Korea) and DNA quality and concentration were assessed via spectrophotometry (NanoDrop 2000; Thermo Fisher Scientific, USA). Sequencing was performed by Bioneer Corp. (Korea). In IGF2 gene, one SNP was discovered in intron 1 (rs315806609, A/G) and a synonymous SNP was discovered in exon 3 (rs313810945, T/C) (Supplementary Figure S1); both SNPs were used for genotyping.

### Next-generation sequencing data analysis

The next-generation sequencing (NGS) data for the KNCs was provided by the National Agricultural Biotechnology Information Center (NABIC), Jeonju, Korea to identify SNPs in the *DUSP8* gene of the KNC-R line. We found six SNPs, including, one missense mutation (rs736096076, G>A) and five synonymous SNPs (rs313158156, C>T; rs313443014, C>T; rs314568020, G>A; rs315155184, G>A; and rs734913846, C>T). The synonymous SNP (rs313443014, C>T) of DUSP8 was selected for amplification and genotyping. This SNP (rs313443014, C>T) of DUSP8 gene is found in the coding sequence (exon 1) leading to the alanine (A), an amino acid used as energy source and is converted into glucose by the liver during the intensive exercise[46].

### Genotyping of *DUSP8* and *IGF2* genes

The PCR restriction fragment length polymorphism (PCR-RFLP) and Kompetitive allele-specific PCR (KASP) methods were used to genotype the *DUSP8* and *IGF2* genes, respectively. For DUSP8, the restriction enzyme *Msp*I was selected using NEBcutter2 software (https://nc2.neb.com/NEBcutter2/) to digest the amplicon (406 bp). The volume of the digestion mixture was 20 μL, containing 15 μL of PCR product, 0.4 μL of restriction enzyme (*Msp*I), 2 μL of 10× CutSmart buffer, and 2.6 μL of deionized distilled water. The mixture was incubated at 37°C, for 12 h. Visualization of the DUSP8 genotypes was performed using a UV transilluminator following 3% agarose gel electrophoresis at 120 V for 30 min. For IGF2 genotyping, SNP target-specific primers were prepared for the KASP genotyping assay (Table 1). The KASP assay mix and KASP master mix (Table 1) were validated and produced by SeouLin Bioscience (Seongnam, Korea) and LGC Genomics Ltd. (Teddington, UK), respectively.

### IMP, inosine, and hypoxanthine analyses using nuclear magnetic resonance

IMP, inosine, and hypoxanthine contents were analyzed using nuclear magnetic resonance (NMR) as described previously [47]. Briefly, the steps of the analysis were chicken sample preparation, polar metabolite extraction, reconstitution of the meat extracts, and NMR data acquisition. The results are expressed as mg/100 g of breast meat. These results were used for an association analysis between DUSP8 and IGF2 genotypes and nucleotide contents.

### Genotype, allele frequencies, and Hardy-Weinberg equilibrium analyses

Genotype and allele frequencies of the *DUSP8* and *IGF2* genes were calculated after genotyping. The genotype frequency was calculated as follows [48]:


(1) $${\text{x}}_{\text{ii}}=\frac{\sum_{\text{i}=1}^{\text{n}}{\text{n}}_{\text{i}}}{\text{N}}$$

The allele frequency was calculated as follows [49]:


(2) $${\text{x}}_{\text{i}}=\frac{\left(2{\text{n}}_{\text{ii}}+\sum_{i\neq j}n_{ij}\right)}{2\text{N}}$$

where x_ii_ is the iith genotype frequency, x_i_ is the ith allele frequency, n_ii_ is the number of samples with the iith genotype, n_ij_ is the number of samples with the ijth genotype, and N is the total number of samples.

Hardy-Weinberg equilibrium (HWE) was calculated as follows [49]:


(3) $$\chi^{2}=\sum_{\text{i}=1}^{\text{N}}\frac{{\left(\text{O}-\text{E}\right)}^{2}}{\text{E}}$$

where O and E are the total numbers of observed and expected genotypes, respectively, and N is the total number of samples.

### Statistical analyses

We used R v4.2.1 software [50] to perform all statistical analyses. Analysis of variance (ANOVA) was performed to assess the effects of the DUSP8 and IGF2 genotypes on the nucleotide contents of the KNC-R line. The homogeneity of variance assumption of the ANOVA was tested using Levene’s test, and the normality assumption was tested using the Shapiro-Wilk test. The association between the DUSP8 and IGF2 genotypes and nucleotide contents was analyzed by a two-way ANOVA for the IMP and hypoxanthine contents and Welch’s one-way ANOVA was used to assess the effects of genotypes and sex on inosine content because the variances were not equal. The following is a 2-way ANOVA model: **Y_i,j,k_** = **μ**+**α_i_**+**β_j_**+**(αβ)_i,j_**+**ɛ_i,j,k_**

Where Y_i,j,k_ is the observation on nucleotide content, μ is the population mean for nucleotide content, α_i_ is the genotype effect, β_j_ is the sex effect, (αβ)_i,j_ is the interaction effect between genotype and sex, and ɛ_i,j,k_ is the residual error.

Tukey’s honest significant difference (HSD) test was used to compare mean values among the genotypes and the p-values (p<0.05) were considered to be statistically significant.

## RESULTS

### *IGF2* sequencing analysis

To identify SNPs in *IGF2* gene, the Sanger sequencing results were analyzed using Bioedit [51]. Sequence analyses of different alleles of the KNC *IGF2* gene were based on a reference sequence from the Ensembl database (https://asia.ensembl.org/; accession no. ENSGALG00000035282). Two SNPs were identified, including one intron variant and one synonymous mutation (Supplementary Figure S1).

### Polymorphism analyses of *DUSP8* and *IGF2* genes

**Figure 1:**
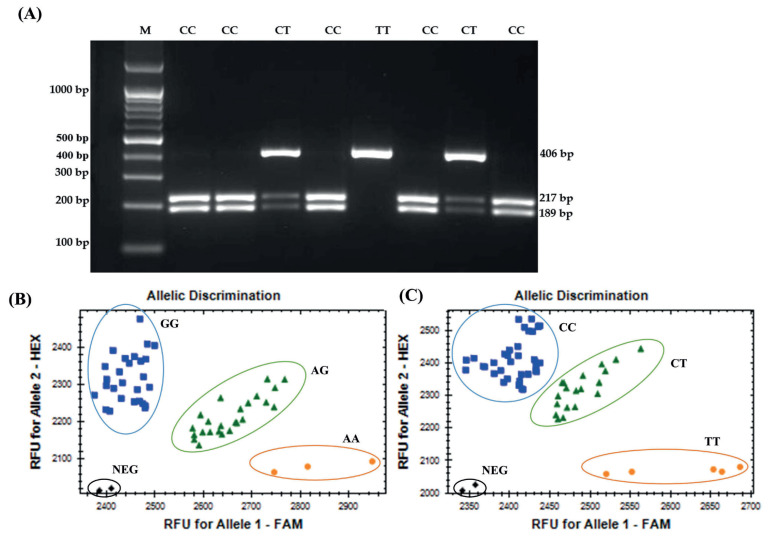
RFLP genotyping results of *DUSP8* gene (SNP rs313443014C>T) visualized on 3% agarose gel electrophoresis from samples 1 to 8 (A); KASP genotyping results for SNP in intron 1(rs315806609A/G) (B); and synonymous SNP (rs313810945T/C) (C) of *IGF2* gene in Korean native chicken-red-brown line (KNC-R line). RFLP, restriction fragment length polymorphism; *DUSP8*, dual-specificity phosphatase 8; KASP, Kompetitive allele-specific polymerase chain reaction; SNP, single-nucleotide polymorphism; NEG, negative control; M, DNA marker with 100 bp.

Genotyping of the *DUSP8* gene was performed using the RFLP method. SNP (rs313443014, C>T) in DUSP8 was located in exon 1 of chicken chromosome 5. The 406 bp fragment (rs313443014, C>T) of the *DUSP8* gene was successfully amplified and its PCR product was digested using *Msp*I, resulting in three genotypes: CC (two fragments of 189 and 217 bp), CT (three fragments of 189, 217, and 406 bp), and TT (an uncut fragment of 406 bp) (Figure 1A–C). These results were visualized by 3% agarose gel electrophoresis. IGF2 was genotyped using the KASP method. KASP genotyping for SNPs in intron 1 (rs315806609, A/G) produced three genotypes: AA, AG, and GG. For the synonymous SNP (rs313810945, T/C), KASP genotyping produced three genotypes: TT, CT, and CC (Figure 1A–C).

### Genotype and allele frequencies of SNPs in *DUSP8* and *IGF2* genes

For *DUSP8* gene, the C allele was the most frequent in the KNC-R line (66%) followed by the T allele (34%) in the total samples. The CT heterozygous genotype was the most frequent (58%), followed by the CC (37%) and TT (5%) genotypes for the total samples (Table 2). The synonymous SNP (rs313443014, C>T) of DUSP8 deviated from HWE (Table 2) for the population A and also in the total sample. For IGF2, in the intron 1 SNP (rs315806609, A/G), the G allele had the highest frequency (72%) among all samples; in the synonymous SNP (rs313810945, T/C), the C allele was the most frequent (75%). In SNP rs315806609 (A/G), the homozygous GG genotype was the most frequent (50%), followed by the AG heterozygous (43%) and the AA homozygous (7%) genotypes. In SNP rs313810945 (T/C), the CC genotype had the highest frequency (57%), followed by CT (36%) and TT (6% (Table 2). The χ^2^ tests showed that both SNPs of the *IGF2* gene (rs315806609, A/G and rs313810945, T/C) in the KNC-R line were in HWE (p<0.05; Table 2).

### Association of SNPs in DUSP8 and IGF2 genotypes with nucleotide contents

We detected a highly significant association (p<0.01) between IMP, inosine, and hypoxanthine contents and SNP rs313443014 (C>T) in exon 1 of the *DUSP8* gene and the intron 1 and exon 3 SNPs (rs315806609, A/G and rs313810945, T/C) of the *IGF2* gene in the KNC-R line (Table 3).

### Sex effects on nucleotide contents

We detected a significant association (p<0.05) between sex and the IMP and hypoxanthine contents in the KNC-R line. Females had a higher IMP content than males, whereas males had a higher hypoxanthine content than females (Table 4).

## DISCUSSION

In this study, we investigated the breeding potential of KNC-R chickens, which have a higher IMP content than other KNC lines [15]. The *DUSP8* and *IGF2* genes were previously investigated through a GWAS as candidate genes for altering nucleotide contents. Both DUSP8 and IGF2 are polymorphic genes in the KNC-R line. DUSP8 has been described as a gatekeeper for glucose disposal to cells [38]; this glucose is broken down to produce ATP, followed by IMP [17,35,45]. Like insulin, IGF2 performs various functions such as glucose usage leading to IMP production [39–42]. IMP is the most abundant nucleotide in meat, and its content has been reported to influence meat flavor [15,52]. Inosine and hypoxanthine were also reported to influence meat aroma [16,17].

Genotyping of the *DUSP8* gene resulted in three genotypes (CC, CT, and TT). Our results show that SNP of the *DUSP8* gene (rs313443014, C>T) in the KNC-R line deviated from HWE for the population A and this influenced the total sample. This deviation from HWE might be attributed to the selection, which lead to the formation of the five lines of KNCs [53]. Large populations can deviate from HWE as a result of selection, mutation, gene flow, and/or genetic drift [28,54,55]. Our results show that chickens with the CC homozygous genotype had higher inosine and hypoxanthine contents than those with the CT genotype, whereas chickens with the CT heterozygous genotype had a higher IMP content than those with the CC and TT genotypes. Thus, DUSP8 has a significant effect on the nucleotide contents in chickens of the KNC-R line.

The χ^2^ tests showed that both SNPs (rs315806609, A/G and rs313810945, T/C) of the *IGF2* gene were in HWE (p<0.05) in the analyzed population. This may be attributed to the absence of evolutionary pressures such as mutation, selection, genetic drift, and gene flow in the population under consideration [28,54,55]. The IGF2 genotypes were significantly associated with IMP, inosine, and hypoxanthine contents. For SNP rs315806609 (A/G), the GG homozygote genotype was significantly associated with higher inosine and hypoxanthine contents than the AG and AA genotypes. By contrast, the AG heterozygous genotype was significantly associated with a higher IMP content. For SNP rs313810945 (T/C), the CC homozygous genotype was significantly associated with a higher inosine content and a lower IMP content. For the latter SNP, there was no significant difference in hypoxanthine content among the three genotypes in 10-week-old chickens; however, the CT heterozygous genotype was significantly associated with a higher IMP content than both homozygous genotypes (Table 3).

Our study also detected effects of sex on nucleotide contents. Female chickens had a higher IMP content than male chickens, whereas males had a higher hypoxanthine content. These results are consistent with those of a previous study that reported a higher IMP content in females than in males [11]. However, there was no significant effect of sex on inosine content (Table 4). IMP content has been reported to be influenced by chicken breed, age, and sex, as well as cooking method [11,14,16]. KNCs contain higher amounts of IMP than broilers and Hinai-jidori (native to Japan), Wenchang and Xianju (native to China), and Lingnanhuang (Chinese commercial broiler line) chickens [18]. Nucleotides affect meat flavor [16,17], which governs meat consumer preferences [18]. Increasing the IMP content in chickens may improve meat flavor and consumer acceptance. Therefore, the discovery of three genotypes among the *DUSP8* and *IGF2* genes that are associated with nucleotide content variation in chickens is highly beneficial as these genotypes may be used in selection and breeding programs to improve chicken meat flavor. In future research, the results of this study should be validated using larger sample sizes and different KNC lines, as well as in different chicken breeds.

## CONCLUSION

In this study, we identified SNPs among the *DUSP8* and *IGF2* genes of the KNC-R line that influence chicken meat nucleotide contents. The heterozygous (CT) genotype of a synonymous SNP (rs313443014, C>T) of the *DUSP8* gene had a higher IMP content, whereas the homozygous genotype (CC) had higher inosine and hypoxanthine contents. For IGF2, a heterozygous (AG) genotype of the SNP rs315806609 (A/G) and a CT genotype of the SNP rs313810945 (T/C) had higher IMP contents than homozygous genotypes, which had higher inosine and hypoxanthine contents. Female KNC-R chickens had a higher IMP content than males, whereas males had a higher hypoxanthine content than females. Therefore, the SNP rs313443014 (C>T) of the *DUSP8* gene and the SNPs rs315806609 (A/G) and rs313810945 (T/C) of the *IGF2* gene may be used as genetic markers in selection and breeding programs to improve the flavor of KNC-R chicken meat.

## Figures and Tables

**Table 1:** Primer information, SNP, PCR product (bp), annealing temperature, and genotyping method for *DUSP8* and *IGF2* genes

Gene	SNP/location	Primer F/R	Amplicon (bp)	Annealing temperature (°C)	Genotyping method
*DUSP8*	Exon 1 (rs313443014C>T)	F: 5′-GTTGTCCTGCCACCTGACTG-3′ R: 5′- GAGACCTTGTCCTGCTGGAG -3′	406	63	RFLP
*IGF2*	Exon 1	F: 5′-CGCTGATACTCCCATGGACC-3′ R: 5′- CCCTCAGAAGGCATCAGACC-3′	1,061	65	
	Exon 2	F: 5′- CTGTGGTCACTGCAGGAGAG-3′ R: 5′-TGCTCGCCCATTTTACAGGT-3′	929	62	
	Exon 3	F: 5′-TGTCAGTAACCCACCTTGTGT-3′ R: 5′-TCAGCGATGGTTTCAAAAAGG-3′	990	62	
	Intron 1 (rs315806609A/G)	Forward primer X, Y (5′-3′) GAAGTTTGTTTTCTGCAATTTCTTTGACTT/AAGTTTGTTTTCTGCAATTTCTTTGACTC Common primer CAGCATGCAGAGCCTTGAAGTGTTT Fluorescent color A/G (FAM/HEX)	-	55	KASP
	Exon 2 (rs313810945T/C)	Forward primer X, Y (5′-3′) AGAGCTTCCAGAAGCCATCTCAT/GAGCTTCCAGAAGCCATCTCAC Common primer GCCACACGTTGTACTTGGAGTACTT Fluorescent color T/C (FAM/HEX)	-	55	KASP

SNP, single-nucleotide polymorphism; PCR, polymerase chain reaction; DUSP8, dual-specificity phosphatase 8; IGF2, insulin-like growth factor 2; F/R, forward and reverse primers; bp, base pairs; RFLP, restriction fragment length polymorphism; KASP, kompetitive allele specific PCR.

**Table 2:** Genotype frequency, allele frequency, and chi-square test results in the *DUSP*8 and *IGF2* genes of the KNC-R line

Gene	SNP	Population	N	Genotype frequency			Allele frequency		χ^2^ calc.
*DUSP8*	rs313443014 C>T	A	79	CC (8) 0.10	CT (70) 0.89	TT (1) 0.01	C 0.54	T 0.46	49.48
		B	205	CC (98) 0.48	CT (93) 0.45	TT (14) 0.07	C 0.70	T 0.30	1.5
		Total	284	CC (106) 0.37	CT (163) 0.58	TT (15) 0.05	C 0.66	T 0.34	22.14
*IGF2*	rs315806609 A/G	A	25	GG (7) 0.28	AG (16) 0.64	AA (2) 0.08	G 0.60	A 0.40	2.78
		B	205	GG (109) 0.53	AG (82) 0.40	AA (14) 0.07	G 0.73	A 0.27	0.08
		Total	230	GG (116) 0.50	AG (98) 0.43	AA (16) 0.07	0.72	0.28	0.46
	rs313810945 T/C	A	25	CC (8) 0.32	CT (14) 0.56	TT (3) 0.12	C 0.68	T 0.32	0.16
		B	205	CC (122) 0.60	CT (71) 0.34	TT (12) 0.06	C 0.77	T 0.23	0.15
		Total	230	CC (130) 0.57	CT (85) 0.36	TT (15) 0.06	G 0.75	A 0.25	0.69

*DUSP8*, dual-specificity phosphatase 8; *IGF2*, insulin-like growth factor 2; KNC-R, Korean native chicken -red-brown line; SNP, single-nucleotide polymorphism; χ^2^ calc.: chi-square calculated; χ^2^ table (p<0.05) = 3.84.

Individuals with specific genotypes are shown in parentheses.

**Table 3:** Association of DUSP8 and IGF2 genotypes with IMP, inosine, and hypoxanthine contents in the KNC-R line

Gene	SNP	Trait	Genotype, *χ̄*±SD		
			CC (n = 106)	CT (n = 163)	TT (n = 15)
			
*DUSP8*	rs313443014 C>T	Inosine	39.12±9.25^a^	34.06±10.91^b^	37.33±9.48^ab^
		Hypoxanthine	6.35±2.08^a^	4.83±2.15^b^	6.00±2.25^ab^
		IMP	176.78±23.30^b^	190.89±25.22^a^	177.61±13.91^b^
	
			GG (n = 116)	AG (n = 98)	AA (n = 16)
	
*IGF2*	rs315806609A/G	Inosine	41.03±9.61^a^	33.42±8.70^b^	32.60±8.80^b^
		Hypoxanthine	6.03±2.34^a^	5.61±2.26^b^	5.27±1.93^b^
		IMP	178.46±23.95^b^	187.00±24.87^a^	182.05±23.05^ab^
	
	rs313810945T/C		CC (n = 130)	CT (n = 85)	TT (n = 15)
	
		Inosine	39.78±9.99^a^	33.38±9.00^b^	32.48±9.10^b^
		Hypoxanthine	6.30±2.26	5.36±2.16	5.21±1.88
		IMP	174.71±21.40^b^	188.31±24.12^a^	183.32±20.16^ab^

Nucleotide contents were expressed as mg/100 g.

Individuals with specific genotypes are shown in parentheses.

*DUSP8*, dual-specificity phosphatase 8; *IGF2*, insulin-like growth factor 2; IMP, inosine-5′-monophosphate; KNC-R, Korean native chicken -red-brown line; SNP, single-nucleotide polymorphism; SD, standard deviation.

a,b Means in the same row with different superscripts are strongly different (p<0.01).

**Table 4:** Effects of sex on IMP, inosine, and hypoxanthine contents in KNC-R line

N1)	Trait	Sex	

		Male (n = 106)	Female (n = 124)
230	Inosine	37.37±9.56	36.56±10.55
	Hypoxanthine	6.96±2.35^a^	4.80±1.68^b^
	IMP	177.20±23.20^b^	186.54±24.35^a^
	
		**Male (n=127)**	**Female (n=157)**
	
284	Inosine	36.17±10.22	36.09±10.75
	Hypoxanthine	6.56±2.44^a^	4.56±1.60^b^
	IMP	181.13±25.23^b^	187.99±24.39^a^

IMP, inosine-5′-monophosphate; KNC-R, Korean native chicken -red-brown line.

1) N, number of samples.

Individuals with specific genotypes are shown in parentheses. Nucleotide contents were expressed as mg/100 g.

a,b Means in the same row with different superscripts are significantly different (p<0.05).

## SUPPLEMENTARY MATERIAL

**Supplementary Figure S1**. Electropherogram Data showing the location of the SNP (rs315806609A/G) located in the intron 1 (A) and synonymous SNP (rs313810945T/C) in the exon 3 of the *IGF2* gene in the KNC-R line (B).
