# Supplementary material for: Association of single-nucleotide polymorphisms in dual specificity phosphatase 8 and insulin-like growth factor 2 genes with inosine-5′-monophosphate, inosine, and hypoxanthine contents in chickens
Source: Anim Biosci. 2023 Jun 23;36(9):1357–66. doi: 10.5713/ab.23.0080 (PMC10472161; doi:10.5713/ab.23.0080)
Supplement: Supplementary file 1 [file ab-23-0080-Supplementary-Fig-1.pdf]

(A) **(B)**

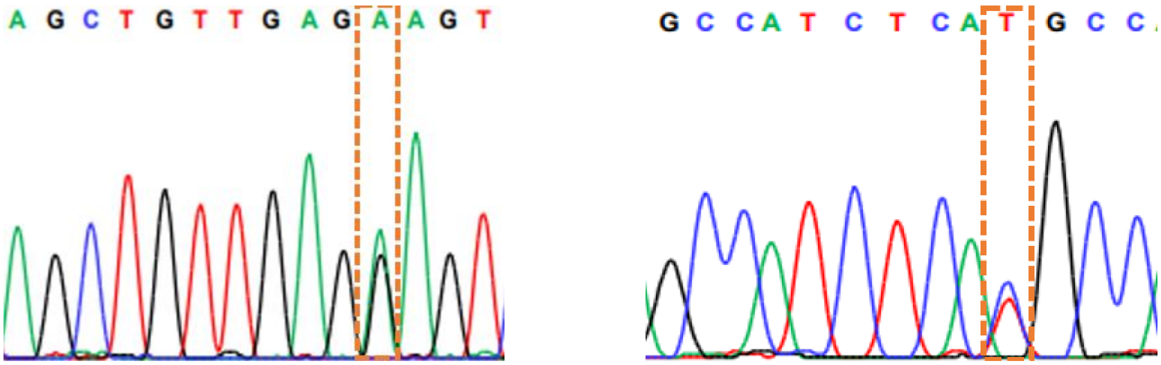

Figure S1. Electropherogram Data showing the location of the SNP (rs315806609A/G) located in the intron 1 (A) and synonymous SNP (rs313810945T/C) in the exon 3 of the *IGF2* gene in the KNC-R line (B).
